# Feasibility of conducting an active exercise prehabilitation program in patients awaiting spinal stenosis surgery: a randomized pilot study

**DOI:** 10.1038/s41598-019-48736-7

**Published:** 2019-08-22

**Authors:** Andrée-Anne Marchand, Margaux Suitner, Julie O’Shaughnessy, Claude-Édouard Châtillon, Vincent Cantin, Martin Descarreaux

**Affiliations:** 1 Department of Anatomy, Université du Québec à Trois-Rivières, Trois-Rivières, Canada; 2 Department of Human Kinetics, Université du Québec à Trois-Rivières, Trois-Rivières, Canada; 3 Department of Chiropractic, Université du Québec à Trois-Rivières, Trois-Rivières, Canada; 4 Centre intégré universitaire de santé et de services sociaux de la Mauricie-et-du-Centre-du-Québec, Trois-Rivières, Canada

**Keywords:** Osteoarthritis, Pain management, Rehabilitation, Surgery, Musculoskeletal system

## Abstract

Prehabilitation is defined as the process of augmenting functional capacity before surgery in preparation for the postoperative phase. This study intends to assess the feasibility of conducting a preoperative intervention program in patients with lumbar spinal stenosis and to report on the piloting of the proposed intervention. Patients were allocated to a 6-week supervised preoperative rehabilitation program or a control group. The intervention included supervised exercise sessions aimed to improve strength, muscular endurance, and spinal stabilization. Outcomes were measured at baseline, 6 weeks later and again 6 weeks, 3 months and 6 months after surgery. Sixty-five percent of admissible participants agreed to take part in the study, of which 5% dropped out before the end of the intervention period. Eighty-eight percent of potential training sessions were delivered without adverse event. Improvements were seen in favour of the experimental group at the preoperative assessment for active ranges of motion, leg pain intensity, lumbar extensor muscle endurance and walking capacities. Results show that slight modifications to the choice of outcome measures would increase feasibility of the main study. The absence of adverse events coupled with positive changes seen in dependant outcome measures warrant the conduct of a full-scale trial assessing the effectiveness of the intervention.

## Introduction

With aging of the North American population, it is expected that the proportion of individuals over 65 years of age which currently represent 15% of the US population (46 million) will rise to 21% in 2030 and almost double in 2060 (98 million)^1^. Similar trends are observed in Canada, with people 65 years of age and older representing 16% of the general population and 19% in the province of Quebec specifically^2^. While, for the first time in history, the proportion of Canadian elderly equals that of children under the age of 14, their health care costs now account for 50% of national public expenses in health care, of which the majority is spent on hospital expenses^3^. As the population continues to grow older, increasing demand is expected on health care systems.

Although Canada performs well when it comes to wait times for priority procedures (ie. cataract, hip and knee), which account for less than 50% of performed elective surgeries, the wait for elective surgery is estimated to be longer in Canada than other developed worldwide countries^4^. As such, despite being one of the most frequent degenerative conditions in older-aged patients^5^ and the main reason for undergoing surgery in adults aged over 65^6^, waiting time for lumbar spinal stenosis (LSS) surgery reaches a median of 140 days from initial consultation with a spine surgeon up to a median of 349 days from primary care physician referral^7^.

Lumbar spinal stenosis is known to cause compression and ischemia of the lumbosacral nerve roots secondary to the narrowing of central and vertebral canals most commonly brought on by degenerative changes including thickening of the articulating facet joints, infolding of the ligamentum flavum and degenerative bulging of the intervertebral discs^8–10^. A dominant feature of LSS is walking limitation due to neurogenic claudication characterized by unilateral or bilateral buttock, thigh or calf pain, discomfort or weakness. Symptoms are triggered by prolonged standing and walking and relieved by bending forward and sitting^11^. Neurogenic claudication causes high levels of disability, leading to a more sedentary lifestyle and loss of independence in the elderly population^12^. Furthermore, inactivity has numerous known deleterious effects on the musculoskeletal and cardiovascular systems that are both central to functional independence^13^. Similarly, there is evidence that older adults undergoing surgery who are physically active, have good nutritional state and adequate mental function have higher levels of functional health and lower postoperative complications^14^. Despite unequivocal benefits of surgical interventions in patient with LSS, results based on pain and disability levels suggest incomplete long-term recovery^15^.

In contrast to rehabilitation, commonly delivered postoperatively, prehabilitation is rooted in the belief that the preoperative period is a salient period to encourage patients to embrace and increase compliance to new healthy habit by educating and preparing them for the tasks that need to be completed in the postoperative period^16^. As such, the concept of prehabilitation is defined as the process of enabling patients to better withstand the stress of surgery, and therefore prompting faster recovery, by augmenting functional capacity and physiological reserve prior to a surgical intervention^13,17^. Indeed, prehabilitation has shown to enable postoperative reduction of pain levels and hospitalization duration, and accelerated return to baseline physical function^18^. Although physiological reserve entails nutritional, metabolic and mental components, a structured exercise program is believed to be the cornerstone of prehabilitation^19^. Generic components of a prehabilitation program include a warm-up, cardiovascular component, resistance exercises and functional training which theoretically would prepare individuals to appropriately handle stresses associated with the surgical procedure^20^. Even though minimal research has been conducted so far regarding prehabilitation within the context of spinal surgery, patients with LSS may be one of the best population to study its effect considering the safety of watchful-waiting in this slowly progressing condition^21^.

Consistent with current evidence stressing the importance of conducting feasibility and pilot studies to identify and subsequently avoid potential problems that may arise in an ensuing randomized control trial^22^, we followed the framework proposed by Eldridge *et al*. (which sates that feasibility is an imbedded component of pilot studies) to report on the feasibility and piloting of the proposed prehabilitation intervention^23^.

Therefore, the main objectives of this study were to (1) assess the feasibility of conducting an active prehabilitation program in patients awaiting decompression surgery for LSS and (2) to report on preliminary results of the piloting of the intervention.

More specifically, for the feasibility component of this study, we aimed to (1) estimate recruitment, attrition and adherence rates, (2) ensure safety of the intervention, (3) test data collection of self-reported outcomes and physical assessment procedures, and (4) assess treatment fidelity. For the piloting component, we aimed to (1) identify if changes occurred in any of the dependant outcomes after the prehabilitation intervention, and (2) identify estimates of variance for sample size calculation for future trial planning.

## Methods

### Study design

The present study was a single-blinded, two-arm randomized pilot trial and its detailed methods have been previously published elsewhere^24^. The trial received ethical approval from the institutional review boards of the Université du Québec à Trois-Rivières (UQTR) and the Centre intégré universitaire de santé et de services sociaux de la Mauricie-et-du-Centre-du-Québec (CIUSSS-MCQ - formerly known as Centre de Santé et de Services Sociaux de Trois-Rivières) (CÉR-2014-008-00). All methods were carried out in accordance with the relevant guidelines and regulations. The trial was registered with the US National Institutes of Health Clinical Trials registry (NCT02258672; October 7th, 2014). We followed the Consolidated Standards of Reporting Trials guideline for randomized controlled trial. Informed written consent was obtained from each participant before any intervention was initiated.

### Participants and setting

All patients were recruited at the Trois-Rivières’ regional hospital (Quebec, Canada) in collaboration with the neurosurgery team. The inclusion criteria were the following: having a clinical history and diagnostic imaging evidence of LSS; having degenerative LSS primarily of central origin affecting one or multiple vertebral levels; awaiting LSS surgery (minimally invasive or open approach); being over 18 years of age; and being able to provide written informed consent voluntarily. Exclusion criteria included presence of non-degenerative LSS, inflammatory arthritic conditions, vertebral instability requiring non-instrumental or instrumented fusion and altered cognitive capacities; individuals deemed ineligible by their treating neurosurgeon; and being unable to understand or express oneself in French.

### Sample size

Sample size determination of N = 40 was guided by time constraints but also by feasibility issues related to patients’ surgery rate conducted over a 1-year period at the recruitment site in accordance with our inclusion and exclusion criteria. Furthermore, when one of the study goals is to obtain an estimate of variance in an outcome when a minimally clinical important difference between groups has already been established (meaning that only the variance needs to be estimated), it is suggested that 10 to 20 participants per group is deemed sufficient to inform feasibility and to plan for a larger study^25^.

### Randomization and minimization

Following baseline evaluation, participants were randomly allocated to one of the two groups. The principal investigator opened an opaque, sealed envelope in front of the participants. The allocation sequence was computer-generated and prepared by a research assistant not involved in the study process. To ensure good balance of prognostic factors in our small sample, four minimization criteria known to delay postoperative recovery were considered (1) presence of diabetes, (2) objective motor deficits in the lower limbs (confirmed by electromyography), (3) self-reported severe disability (Oswestry Disability Index score ≥41%) and (4) smoking habit.

### Blinding

Participants were not blinded to intervention allocation, but content of exercise sessions was known only to those in the intervention group to prevent cross-contamination between groups. The kinesiologist involved in the training of participants did not take part in the evaluation sessions as the main investigator conducted all evaluations and follow-ups.

### Intervention

#### Control group

Patients followed the regular hospital preoperative management and received, the day prior to surgery, standardized written information on how to keep a good back posture when getting in or out of bed and when sitting down.

#### Intervention group

**Figure 1:**
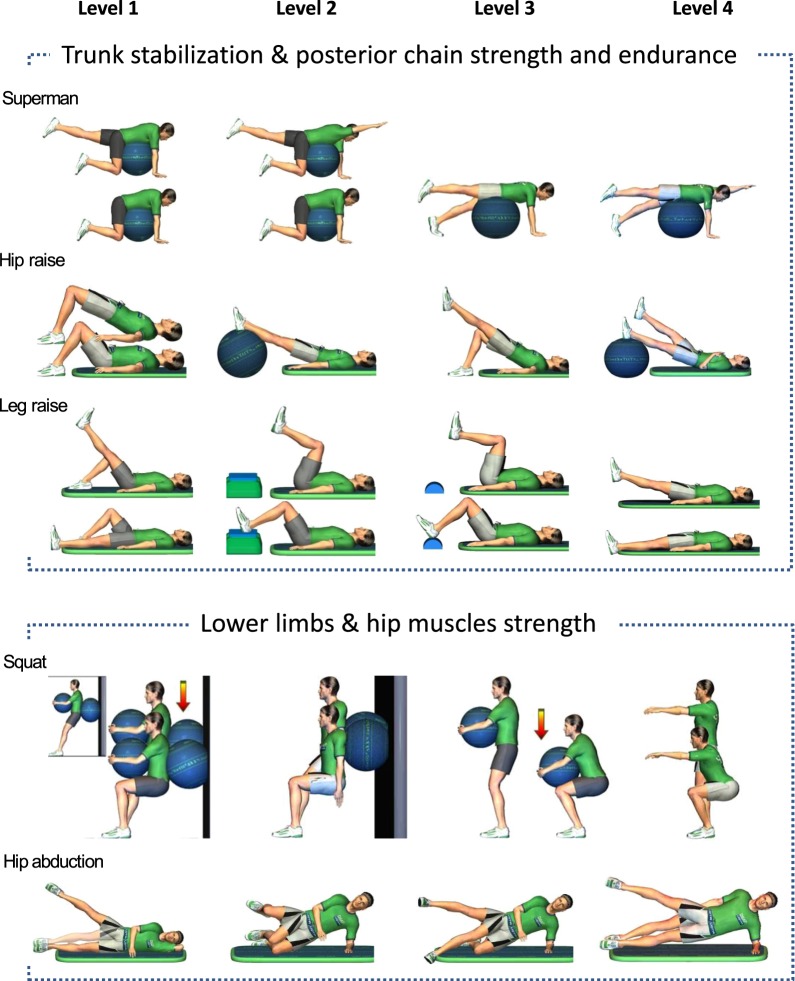
Exercise progression for the intervention group.

Patients received an exercise-based intervention 3 times per week for 6 weeks prior to their surgery. Training sessions were individually supervised by a certified kinesiologist and lasted 30 minutes. Sessions began with a 5-minute warm-up which consisted of cycling (stationary) or walking (treadmill) based on participants’ preference, followed by five muscular exercises with concentric or isometric phases that aim to improve muscle and structures involved in walking capacities. Each exercise intensity level was tailored to the participant’s capacity and progressively modified to obtain increasing levels of difficulty in order to provide a safe, individualized and yet motivating training experience for each participant. Exercises and their progression are detailed in Fig. 1. The intervention was performed at the Université du Québec à Trois-Rivières. For each patient, the kinesiologist used a logbook to document, for each exercise, the number of repetitions and levels of difficulty reached, perceived effort and discomfort if any, including location, intensity and character. Patients in the intervention group received the same standardized written information as the control group.

### Data collection

**Figure 2:**
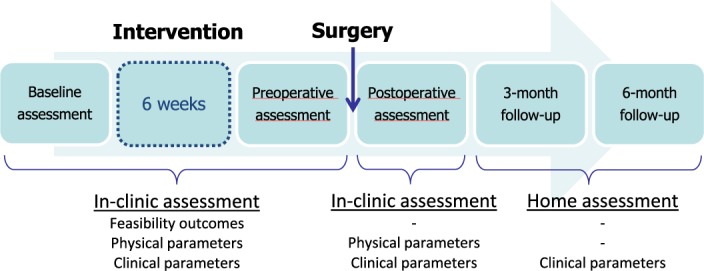
Timeline of intervention and assessments.

Questionnaire-based patient-reported outcome measures were collected at UQTR’s research facility at baseline, after 6 weeks prehabilitation intervention, and 6 weeks post-surgery, and by mail at 3- and 6-months post-surgery. Physical outcome measures were collected at UQTR’s research facility at baseline, after the 6-week prehabilitation intervention, and 6 weeks post-surgery. A timeline illustrating the intervention and outcome assessments is presented in Fig. 2.

### Primary outcome measures

#### Feasibility component

Feasibility parameters included recruitment, attrition and adherence rates as well as safety of the intervention. The recruitment rate was determined by the number of individuals who participated in the study out of those who were admissible and contacted by phone but refused to participate. Attrition was defined by the number of individuals who gave consent to participate in the study but dropped out before the end of the intervention period, regardless of group allocation. Adherence to the intervention was measured by using the number of exercises sessions delivered out of the theoretical number that could have been delivered based on the surgical wait time of each participant. Safety of the intervention was determined based on the number and nature of adverse event, which was defined as symptoms flare-ups that would prevent a patient from taking part in subsequent training sessions or injuries requiring medical attention. In addition to the kinesiologist taking note of any undesirable effect during in-clinic visits, all participants were asked to report any reaction or flare-up that was not consistent with their usual pain presentation as a result of either exercise or assessment sessions.

#### Piloting components

Self-reported leg pain intensity was measured using an 11-point Numerical Rating Scale^26^. As one of the most important outcome for patients with LSS is to decrease leg pain intensity^27^, the intervention preliminary effects on this variable will be used to estimate adequate sample size for future research planning.

### Secondary outcome measures

#### Piloting components - clinical outcome measures

Preliminary effect of the intervention was investigated using valid clinical outcomes, previously used in LSS studies and deemed relevant to the LSS patients. Patient-reported outcomes were used to document current low back pain intensity (11-point Numerical Rating Scale)^26^, low back disability (Oswestry Disability Index)^28^, quality of life (EuroQol-5D)^29^, and perception of treatment effect (Patient Global Impression of Change). Satisfaction with the proposed intervention and the overall surgical results was measured using a scale of 0 to 100%. Moreover, pain medication intake was measured with daily self-reported journal and hospital chart. Additional clinical information was collected throughout the study, regarding factors considered as potential predictors of intervention response, such as fear avoidance behavior (Tampa Scale of Kinesiophobia)^30^, and level of anxiety and depression (Beck Disability Index)^31^. Similarly, the satisfaction related to work conditions was measured using the Minnesota Satisfaction Questionnaire^32^ during baseline assessment only, for those employed or on sick leave at the time of the study. Lastly, perioperative data including blood loss, length of surgery, surgical technique used, intraoperative complications, and length of hospital stay were documented as potential explanatory factors of between group differences in participants’ status.

#### Piloting components - physical outcome measures

Objective physical outcome measures were used to measure change on participants’ physical condition after the prehabilitation program. Physical tests included lumbar extensor muscles endurance (modified Sorensen test), trunk flexor and extensor muscle strength (isometric contraction), knee extensor muscle strength (isometric contraction), active lumbar ranges of motion, walking abilities (time to first symptoms and total ambulation time), and maximal aerobic capacity. Study protocol provides further information about the selected outcomes^24^.

### Statistical methods

#### Feasibility components

Feasibility outcomes are reported using descriptive statistics (N; %) or qualitative data wherever appropriate.

#### Piloting components

Comparison of between-group demographic data are reported using the independent Student t-test for continuous variables and the chi-square test for categorical variables.

General trends and preliminary assessment of outcomes variability over time were explored using mixed model ANOVAs for group comparison. A priori contrasts were conducted whenever significant main or interaction effects were present. Whenever baseline variables did not follow normal distribution, appropriate transformations were applied in order to conduct parametric statistics. Analyses of clinical and physical outcomes were conducted according to the intention-to-treat principle with participants analyzed according to randomly assigned treatment group irrespective of compliance. Both complete case and imputation analyses were performed. Missing data (mean number = 9.3% per table) were replaced using Winer *et al*.’s least squares imputation method with iteration, subtracting 1 degree of freedom from the interaction term per imputed value^33^. Analyses were computed using Statistica 10 (Statsoft, Tulsa, OK). The level of significance was set to 0.05.

## Results

### Participants

**Figure 3:**
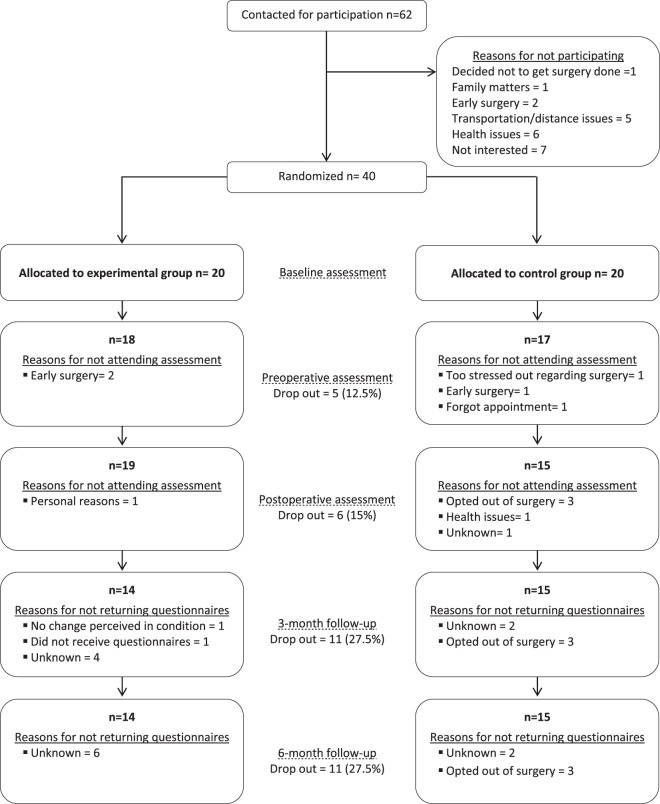
CONSORT flowchart of the pilot trial.

Between February 2015 and June 2016, a total of 62 eligible patients were contacted, of whom 40 agreed to participate and were randomly assigned to the intervention (n = 20) or control group (n = 20) (Participants flow chart is presented in Fig. 3). There was no significant difference between the groups with respect to baseline characteristics except for the maximum isometric lumbar strength in extension which was higher in the intervention group (Table 1).

**Table 1:** Participants’ baseline characteristics.

	Intervention (N = 20) Mean ± SD	Control (N = 20) Mean ± SD	*p*
**Demographics**			
Age – yrs	66.7 ± 11.6	71.5 ± 7.3	0.12
Gender, female – n (%)	9 (45)	8 (40)	0.74
Weight – kg	76.68 ± 16.43	83.11 ± 14.79	0.21
Height – cm	168.25 ± 10.12	162.78 ± 9.28	0.10
**Employment situation – n (%)**			0.54
Currently working	1 (5)	2 (10)	
Sick leave or retired due to pain	9 (45)	3 (15)	
Retired unrelated to pain	10 (50)	15 (75)	
^†^Work satisfaction - /100	85.5 ± 7.7	87.5 ± 12.0	0.43
**Clinical parameters**			
**Pain intensity –/10**			
Back	5.0 ± 3.3	5.9 ± 2.7	0.37
Leg	7.3 ± 2.1	6.5 ± 2.7	0.29
Leg pain dominant – n (%)	15 (75)	13 (65)	0.49
Weekly days with pain – (/7)	6.9 ± 0.2	7.0 ± 0	0.33
Back disability – /100	38.8 ± 16.5	39.5 ± 13.5	0.88
Kinesiophobia – /68	47.6 ± 8.0	45.4 ± 7.3	0.36
Depression – /63	4.2 ± 4.1	5.6 ± 4.6	0.30
**Physical parameters**			
**Lumbar active ROMs – degrees**			
Flexion	61 ± 28	66 ± 21	0.50
Extension	15 ± 8	16 ± 5	0.63
Left lateral flexion	14 ± 7	12 ± 5	0.25
Right lateral flexion	16 ± 8	14 ± 6	0.51
**Trunk muscles strength – N·m**			
Flexion	46.81 ± 23.19	44.79 ± 31.47	0.82
Extension	40.73 ± 31.85	22.76 ± 22.02	0.04*
Knee extensor strength – lbs	56.93 ± 30.6	56.48 ± 30.9	0.96
Lumbar extensor endurance – sec	38.46 ± 52.7	19.97 ± 22.6	0.15
**Walking capacities – sec**			
Time to 1^st^ symptoms	121 ± 101	101 ± 83	0.49
Total ambulation time	189 ± 99	183 ± 160	0.89

*Statistically significant difference between groups; ^†^results based on 2 participants from the intervention group and 2 from the control group.

### Feasibility components

*Recruitment rate* reached 65% with the main reasons for non-participation being lack of interest (N = 7; 32%), concomitance of health issues believed to prevent taking part in a physical training program or overall self-perceived poor physical condition (N = 6; 27%), and transportation difficulties such as being unable to drive by oneself or living in a distant city (N = 5; 23%).

*Attrition rate* at the preoperative assessment was 5% with two participants from the intervention group dropping-out for reasons unrelated to the proposed exercises. Two weeks before the end of the program, one started to experience bouts of increased leg pain known to happen periodically and did not want to exercise through pain (completed 11/18 training sessions). The other one had vacations planned and did not want to risk decreasing the effects of a cortisone injection she was about to receive (completed 2/18 training sessions). No participants from the control group dropped-out before the preoperative assessment.

#### Adherence to the protocol

A total of 8 participants completed all 18 training sessions as planned (40% compliance) whereas 9 completed more than 50% of sessions (range: 11–17) and 3 less than 50% (range: 2–7). Considering that the intervention period was shortened for some participants due to the variable rate of surgical operation for elective surgeries, we can consider that a maximum of 326 sessions could be provided to participants yielding a compliance rate of 88% (288/326). Main reasons for not completing all training sessions, aside from early surgery and the ones mentioned for attrition included lack of transportation, funeral, medical appointments and headache episodes related to a concussion.

#### Adverse event

No adverse events were reported as a result of the training program or physical assessments at any point in time.

#### Testing of data collection procedures - Excluded outcomes

Following completion of the study, outcomes related to cardiovascular capacity and pain medication intake were deemed uninterpretable for this specific sample of LSS population. Out of 40 participants, 21 presented with high blood pressure managed with beta-blockers which mode of action is to limit increase in heart rate. Consequently, heart rate plateaued during the early stage of the test, making it impossible to attain the 85% of maximal capacity required to proceed to linear extrapolation in order to compute VO^2^max values. Furthermore, in participants not reporting hypertension, low physical capacities prevented 14 of them to take part in the cardiovascular evaluation, leaving only 5 participants being able to complete the test. Furthermore, participants took their pain medication as prescribed; regardless of pain intensity both before and after the operation in fear of seeing the symptoms reappear or increase.

#### Treatment fidelity

Treatment fidelity pertaining to the treatment integrity was assessed^34,35^. The process of defining the components of the training sessions was undertaken by three clinical experts familiar with the LSS population but working in different fields (kinesiology, chiropractic and neurosurgery). Clinicians met to agree on a treatment protocol based on current scientific evidence and decided on a set of standardised exercise progression. One certified kinesiologist having a background with the elderly population was employed to provide the intervention to study participants. Initial training of the therapist included discussion on the pathophysiological process and clinical manifestations of LSS with research team members, review of the exercises proper execution and their progression as well as familiarisation with data recording using the study logbook. One study coordinator accompanied the kinesiologist during the first few visits and acted as an external observer to ensure that treatment was delivered as planned and offered feedback when needed. The only reasons that required temporary modifications to the standard procedure were back pain when lying down during the leg raise exercise (n = 7), the inability to kneel on the floor because of knee pain during the superman exercise (n = 2), and loss of balance during the squat exercise (n = 1).

### Piloting components

#### Changes in clinical and physical outcomes after the intervention

**Figure 4:**
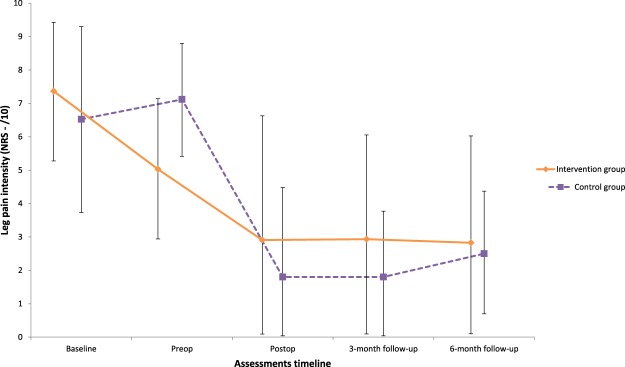
Leg pain intensity.

**Figure 5:**
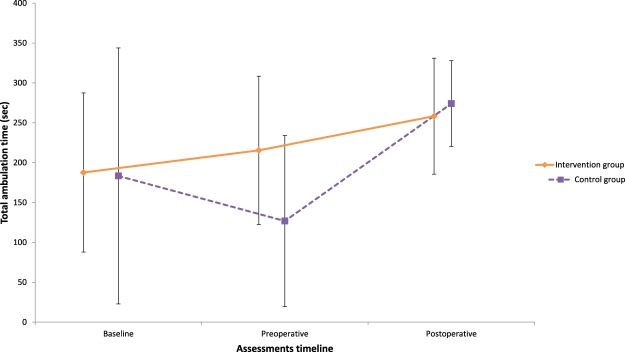
Total ambulation time.

**Figure 6:**
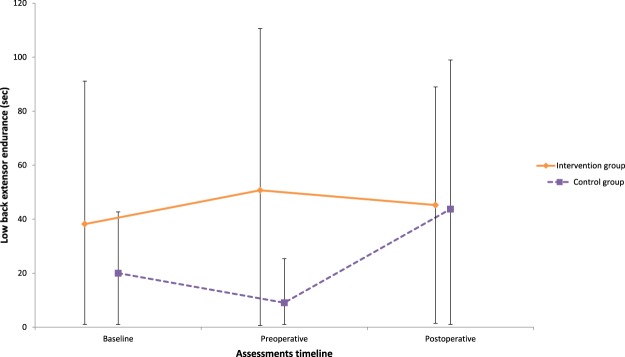
Low back extensor muscles endurance.

Case complete and imputation analyses yielded similar results for the primary outcome. Imputed data analyses are presented from here on. Significant Group x Time interactions were found between the baseline and the preoperative assessment in favor of the intervention group for active lumbar extension (F_2,53_ = 6.29 p = 0.003, ηp^2^ = 0.12) and flexion (F_2,62_ = 5.01, p = 0.009, ηp^2^ = 0.08) ranges of motion; leg pain intensity (F_4,114_ = 6.95, p = p < 0.001, ηp^2^ = 0.06, Fig. 4); total ambulation time (F_2,62_ = 4.08, p = 0.02, ηp^2^ = 0.05, Fig. 5); and low back extensor muscles endurance (F_2,62_ = 5.76, p = 0.05, ηp^2^ = 0.09, Fig. 6). These differences were not maintained at the postoperative and follow-up assessments. Secondary covariance analyses were conducted on the trunk muscles strength in extension variable with adjustment made for baseline values. A significant difference was found in favor of the intervention group at the preoperative assessment (p < 0.001).

Numerous variables improved in both groups overtime, including depression and anxiety (F_4,118_ = 19.1, p < 0.001), low back pain intensity (F_4,114_ = 18.1, p < 0.001), trunk muscles strength in flexion (F_2,62_ = 4.9, p = 0.009), low back related disability (F_4,116_ = 40.6, p < 0.001), and kinesiophobia (F_4,118_ = 16.1, p < 0.001). Results are presented by group based on clinical and physical outcomes for all time point in Tables 2 and 3 respectively.

**Table 2:** Results for clinical outcome measures.

	Group	N	Baseline		N	Preoperative		N	Postoperative	
			Mean ± SD	95% CI		Mean ± SD	95% CI		Mean ± SD	95% CI
NRS back pain/10	Int	20	5.1 ± 3.3	(3.5–6.6)	18	4.6 ± 2.3	(3.4–5.7)	19	2.7 ± 2.3	(1.6–3.8)
	Control	20	5.9 ± 2.7	(4.6–7.2)	17	5.6 ± 2.7	(4.3–7.0)	15	3.4 ± 2.8	(1.8–5.0)
NRS leg pain/10	Int	20	7.4 ± 2.1	(6.4–8.3)	18	5.0 ± 2.1	(3.9–6.1)	19	2.9 ± 3.7	(1.1–4.7)
	Control	20	6.5 ± 2.8	(5.2–7.8)	17	7.1 ± 1.7	(6.3–8.0)	15	1.8 ± 2.7	(0.3–3.3)
Back disability/100	Int	20	38.8 ± 16.5	(31.1–46.6)	18	37.2 ± 15.3	(29.6–44.8)	19	20.4 ± 15.4	(13–27.8)
	Control	20	39.5 ± 13.5	(33.2–45.8	17	40.4 ± 14.7	(32.8–48.0)	15	15.5 ± 15.9	(6.7–24.4)
Kinesiophobia/68	Int	20	47.6 ± 8.0	(43.8–51.4)	18	46.1 ± 7.1	(42.6–49.6)	19	39.8 ± 8.8	(35.5–44.1)
	Control	20	45.4 ± 73	(42.0–48.8)	17	48.3 ± 7.6	(44.4–52.2)	15	38.6 ± 9.6	(33.3–43.9)
Depression/63	Int	20	4.2 ± 4.1	(2.3–6.1)	18	4.5 ± 5.1	(2.0–7.0)	19	2.7 ± 3.7	(0.9–4.5)
	Control	20	5.7 ± 4.6	(3.5–7.8)	17	5.8 ± 5.9	(2.8–8.9)	15	2.3 ± 3.6	(0.3–4.3)
			**3-month**			**6-month**				
		**N**	**Mean ± SD**	**95% CI**	**N**	**Mean ± SD**	**95% CI**			
NRS back pain/10	Int	14	1.8 ± 1.2	(1.0–2.5)	14	2.9 ± 2.9	(1.3–4.6)			
	Control	15	2.8 ± 2.4	(1.5–4.1)	15	3.5 ± 2.3	(2.4–4.8)			
NRS leg pain/10	Int	14	2.9 ± 3.1	(1.1–4.7)	14	2.8 ± 3.2	(0.9–4.6)			
	Control	15	1.8 ± 2.0	(0.7–2.9)	15	2.5 ± 1.9	(1.4–3.6)			
Back disability/100	Int	14	20.3 ± 13.7	(12–28.6)	14	16.4 ± 19.0	(5.9–27.0)			
	Control	15	23.5 ± 13.5	(16.0–31.0)	15	22.9 ± 12.9	(16.0–29.8)			
Kinesiophobia/68	Int	14	39.4 ± 7.9	(34.8–44.0)	14	37.5 ± 7.5	(33.3–41.6			
	Control	15	41.1 ± 7.8	(36.8–45.5)	15	40.7 ± 10.5	(35.2–46.1)			
Depression/63	Int	14	2.7 ± 2.6	(1.2–4.2)	14	2.1 ± 2.2	(0.8–3.4)			
	Control	15	4.2 ± 4.6	(1.6–6.8)	15	3.5 ± 3.5	(1.7–5.3)			

Int = intervention; N = number of cases; SD = Standard Deviation; CI = Confidence Interval; NRS = Numerical Rating Scale.

**Table 3:** Results for physical outcome measures.

	Group	N	Baseline		N	Preoperative		N	Postoperative	
			Mean ± SD	95% CI		Mean ± SD	95% CI		Mean ± SD	95% CI
**Trunk muscles strength N·m**										
Flexion	Int	20	46.8 ± 23.2	(36.0–57.7)	18	55.1 ± 28.1	(40.7–69.5)	19	51.5 ± 29.0	(37.5–65.5)
	Control	20	44.8 ± 31.5	(29.6–60.0)	17	43.7 ± 29.5	(28.0–59.4)	15	47.6 ± 26.4	(33.0–62.2)
Extension	Int	20	40.7 ± 31.9	(25.8–55.6)	18	74.8 ± 69.4	(39.1–110.5)	19	45.2 ± 47.9	(22.0–68.3)
	Control	20	22.8 ± 22.0	(12.1–33.4)	17	29.1 ± 25.2	(15.7–42.5)	15	40.1 ± 39.1	(18.4–61.7)
**Lumbar active ROMs (degrees)**										
Flexion	Int	20	61 ± 28	(48–74)	17	66 ± 22	(54–78)	19	68 ± 19	(58–77)
	Control	20	66 ± 21.9	(56–77.0)	16	61 ± 27	(46–75)	15	74 ± 14	(66–82)
Extension	Int	20	15 ± 8	(10–19)	17	18 ± 6	(14–22)	19	18 ± 7	(14–21)
	Control	20	16 ± 5	(13–19)	16	13 ± 7	(9–17)	15	15 ± 6	(12–19)
Left lateral flexion	Int	20	15 ± 7	(11–18)	17	17 ± 8	(13–22)	19	14 ± 5	(11–17)
	Control	20	12 ± 5	(10–15)	16	12 ± 5	(9–14)	15	14 ± 5	(11–17)
Right lateral flexion	Int	20	16 ± 8	(12–20)	17	16 ± 7	(13–20)	19	14 ± 6	(11–17)
	Control	20	14 ± 6	(11–17)	16	12 ± 5	(9–15)	15	17 ± 6	(13–21)
Knee extensors strength – lbs	Int	20	56.9 ± 30.6	(42.6–71.3)	18	64.1 ± 33.8	(45.3–82.8)	17	63.3 + 39.7	(42.8–83.7)
	Control	20	56.5 ± 30.9	(42.0–71.0)	16	53.5 ± 36.5	(34–72.9)	15	58.4–30.4	(41.5–75.2)
Lumbar extensors endurance – sec	Int	20	38.5 ± 52.7	(13.8–63.1)	17	50.9 ± 59.8	(20.2–81.6)	18	45.5 ± 43.6	(23.8–67.1)
	Control	20	20.0 ± 22.7	(9.4–30.6)	16	9.0 ± 16.4	(0.3–17.7)	15	43.7 ± 55.2	(13.2–74.3)
**Walking capacities – sec**										
Time to 1st symptoms	Int	20	121.4 ± 101.2	(74–168.7)	17	140.5 ± 98.3	(90.0–191.1)	19	208.4 ± 120.2	(150.5–266.4)
	Control	20	101.0 ± 83.1	(62.1–139.9)	16	61.3 ± 59.0	(29.8–92.7)	15	186.4 ± 128.9	(115.0–257.8)
Total ambulation time	Int	20	189.1 ± 99.6	(142.5–235.6)	17	216.8 ± 92.8	(169.1–264.5)	19	259.6 ± 72.7	(223.4–295.7)
	Control	20	183.4 ± 160.6	(108.2–258.5)	16	126.8 ± 107.4	(69.6–184.0)	15	274.1 ± 53.7	(244.3–303.8)

Int = intervention; N = number of cases; SD = Standard Deviation; CI = Confidence Interval; ROM = Ranges of Motion.

Changes in quality of life were considered by comparing proportions of patients reporting improvement in each of the 5 dimensions for each assessment. No between group significant difference was found at any time point (all ps>0.05).

### Lumbar extensor muscles endurance

Imputation of missing data was not possible for lumbar extensor muscle endurance due to the significant number of participants (10/40) who were unable or unwilling to perform the task. For those who achieved a 20% improvement in endurance time intervention group (6/20) compared to (1/20) for the control group p = 0.03 at the preoperative assessment. Results were no longer significant (p = 0.72) at the postoperative assessment (5/20 and 6/20 for the intervention and control group respectively).

*Perceived change in global status* was described as more favorable (p < 0.001) in the intervention group (mean ± SD: 2.8 ± 1.2) compared to the control group (4.4 ± 1.1) at the preoperative assessment. In the intervention group 61% reported “improvements” (= very much better, much better, or slightly better) compared to 20% in the control group. Five percent reported “worsening” (= very much worse, much worse, or slightly worse) in the intervention group compared with 33% in the control group.

#### Satisfaction

Satisfaction (mean ± SD) regarding the intervention program was rated at 93.7% ± 9.1 by 19 of the participants. One participant did not provide a satisfaction score given its short participation duration.

Satisfaction rates regarding postoperative outcomes were similar in both groups with results (mean ± SD) for back pain reaching 83.7% ± 25.9 in the intervention group and 80% ± 25.3 in the control group (p = 0.68). Similarly, satisfaction rate for the postoperative results regarding leg pain reached 84.4% ± 20.9 in the intervention group and 83.1% ± 26.5 in the control group (p = 0.87).

### Intraoperative data

No between group differences were found regarding intraoperative variables and length of hospital stay. Results are presented in Table 4.

**Table 4:** Perioperative data.

	Intervention (n = 20) Mean ± SD	Control (n = 17) Mean ± SD	*p*
*Reported physical activity at the preoperative assessment (n)	7	6	0.73
Length of surgery (min)	108.9 ± 56.7	109.7 ± 75.37	0.97
Blood loss (ml)	120.0 ± 141.8	220.6 ± 269.2	0.15
Intraoperative complication (n)	0	1	
Length of hospital stay (days)	3.6 ± 4.0	4.2 ± 2.6	0.61
Minimally invasive approach (n)	5	2	0.30
Open approach (n)	15	15	1
Received physiotherapy postoperatively (n)	2	2	1

*Data provided based on n = 20 per group. Types of physical activity included treadmill or outdoor walking, stationary or outdoor bicycling, fall risk prevention program, and performing the prehabilitation exercise on off days. Physiotherapy consisted on one hospital-based or home-based visit to ensure adequate independency. The intraoperative complication was a dural tear.

### Sample size estimate

Based on effect size estimate derived from the leg pain intensity data, and considering a significance level of p = 0.05, a power of 90%, and a 20% attrition rate an estimated 58 total patients would be required to detect significant between-group differences. This estimate will be used in a subsequent full-scale randomized trial assessing the effectiveness of the prehabilitation intervention.

## Discussion

One of the main objectives of the present study was to assess the feasibility of conducting an active prehabilitation program in patients with lumbar spinal stenosis. Results showed that adherence and attrition rates were satisfactory, and no adverse event was reported, suggesting that prehabilitation is both feasible and well tolerated despite commonly observed symptoms’ fluctuation in patients with LSS. Another aim was to report on the piloting of the intervention. Preliminary testing of intervention effects showed positive changes in both clinical and physical parameters at the preoperative assessment in favour of those in the prehabilitation group. However, at the postoperative assessment and follow-ups, these differences had leveled out. Most outcomes showed similar trend with improvements seen at the preoperative assessment in favor of the intervention group while the control group remained stable or worsened over time, which reflects the potential added benefits of participating in prehabilitation in the context of spine surgery. In addition, outcomes that were clinically and statistically significant were the ones deemed important by the participants, namely leg pain intensity and walking abilities^27^.

Perceived low health status and transport logistic were reported as the main obstacles for not taking part in the study. In-home telerehabilitation has been reported to yield similar results in patients after total knee arthroplasty compared to face-to-face rehabilitation^36^. Such alternative should be considered in patients that show good execution of the proposed exercises. Poor self-perceived health and high pain intensity have been associated with kinesiophobia in individuals over 65 years of age^37^. In this sample of LSS patients, walking was considered the most common form of physical activity one would undertake if in a pain free context. Given that all participants had limited walking capacities based on the nature of their condition, and that very few of them had continued taking part in social activities (i.e golf, bowling), they tend to refer to walking as the sole potential physical activity when answering the Tampa Scale of Kinesiophobia. Not surprisingly, fear avoidance scores were relatively high and may explain the belief that if one has a hard time walking, which is related to as a basic everyday activity, undertaking any other form of more complex physical activity may appear unrealistic. Providing in depth information to patients regarding how LSS affects their body and means to get round walking incapacities while staying active may open the door to more effectively engaging older patients in improving physical capacities. No adverse event was reported as a result of participation in the present study. However, it can be expected that most patients with low physical fitness would experience temporary delayed onset muscle soreness, at least following the first few exercise sessions. One could argue that in clinical settings even such benign and transient adverse event, may discourage participation in prehabilitation programs. The lack of observed adverse event also emphasizes the importance of proper supervision of participants to ensure appropriate execution of movements especially in the early phase of exercise implementation.

A possible reason that explain the lack of between group differences at the postoperative assessment might be that the major positive impact brought on by the surgery outweighs that of the prehabilitation program. Furthermore, prehabilitation may only have a significant postoperative impact on recurrence of symptoms later down the road rather than immediate benefits. A longer follow-up period should be included in future studies assessing the effectiveness of prehabilitation to allow a better understanding of its potential short and long-term effects. Similarly, a better documentation of concomitant painful conditions, especially those affecting the lower back region and lower limbs that have the potential to influence patients’ postoperative physical performances and clinical status seems warranted. Finally, participants in the control group may have had less fear of performing physical tests after the surgical intervention compared to the preoperative period, although this was not reflected in the kinesiophobia scores. Preliminary results of effectiveness should be interpreted with caution as the study was not adequately powered to answer hypothesis testing.

Very few published studies have reported on the effect of prehabilitation within the context of spine surgery. Nielsen *et al*.^38^ looked at the impact of combined prehabilitation and early rehabilitation in patients awaiting elective spine surgery for degenerative disease that included both low back and radiating pain. The intervention consisted in a 6 to 8 weeks daily individualized home training program, supplemental food intake and early in-hospital rehabilitation. The intervention group reported increased function at the time of operation and recovered and left hospital significantly earlier than the hospital standard care group. In addition, a greater proportion of patients (53%) in the intervention group reported being “very satisfied” regarding the overall treatment (compared to 21% for the control group). Although it is not possible to tease out the effectiveness of each intervention components from the Nielsen study, those results may suggest that a multimodal prehabilitation program is more beneficial in improving immediate postoperative recovery. More recently, Lindbäck *et al*.^39^ reported on the effectiveness of a 9-week presurgery physiotherapy program performed bi-weekly combined with a behavioral approach to reduce fear avoidance and increase activity level. The physiotherapy group was significantly improved after receiving the intervention with between group differences found in low back disability, back pain intensity, quality of life, fear avoidance, self-efficacy and depression. Although they did include degenerative lumbar spine disorders (including disc herniation, spondylolisthesis, degenerative disc disease) 65% of the sample had received a diagnosis of LSS. Follow-ups at 3 and 12 months post-surgery did not show significant group difference except for a larger proportion of patients in the physiotherapy group reporting higher physical activity level at the 1-year follow-up. Similar to the Nielsen’s study and ours, patients in the physiotherapy group were more likely to report improvements than patients in the waiting-list group after the presurgery intervention (49% compared to 17%).

The lack of difference observed between the two groups at the preoperative assessment for some of the outcomes may be explained by the short duration of the intervention. Although a longer intervention could be thought of as ideal to test the full extent of the responses to the intervention, reality is that once patients have opted for surgery, the length of the preoperative delay becomes unpredictable. Similarly, given that physical changes may only be observed over longer period of time it was deemed necessary to add simpler functional tests that would better reflect patients’ activities of daily living. As such, the full scale randomized controlled trial will include the 30 second sit-to-stand^40^ and the timed up and go^41^ tests. These tests will allow for the measurement of progress regarding balance, sit to stand, and walking capacities. In addition, due to the fact that no self-reported outcome measures specific to lumbar spinal stenosis is available in French we instead used outcomes commonly reported in the low back pain literature to measure the clinical incapacities. Although the walking section of the ODI has been shown to be highly correlated to objective walking distance^42^, the participants in our sample found that the questionnaire did not completely capture their daily challenges. As a matter of fact, little changes were observed for the total ODI score throughout the study period despite global perceived change. This observation highlighted the need for a French adaptation and validation of the Swiss Spinal Stenosis questionnaire original version^43,44^.

In light of the limited evidence available regarding prehabilitation within the context of spine surgery, the strengths and limitations of the study should be discussed. The population awaiting surgery was homogenous and the proposed intervention was tailored to individual participants’ physical capacities. One kinesiologist delivered all exercise sessions which limits inter-individual variation and a potential clinician effect. The study used a pragmatic approach echoing the realities of the Quebec’s public health care system which was reflected in the variability of the surgical waiting-list length. If the program was to be offered as part of the public health care regimen it is expected that not all patients would be able to attend a predetermined number of training sessions, therefore prompting the importance to document the effects of the intervention based on different length of time. Patients came from different geographic regions within the province of Quebec, making the results generalizable to provinces with similar health care system. From a methodological perspective, imputation was used to deal with missing data, allowing to preserve reasonable power when conducting the statistical analyses. On the other hand, the principal investigator was aware of the participants’ group allocation when conducting assessments, potentially introducing bias as the assessor may have acted differently with participants form the intervention group than with those from the control group.

## Conclusion

The main objectives of the present study were to assess the feasibility of a 6-week preoperative exercise program in patients awaiting elective surgery for LSS and to report on the preliminary effects of the intervention. Our findings suggest that it is both feasible and safe to train a population presenting a deconditioned physical status, combined with severe pain and disability. Minor changes in the choice of outcome measures are warranted to improve the feasibility for a larger randomized clinical trial. The graded individual exercise program seems to have a beneficial effect on decreasing leg pain intensity, and increasing active lumbar ranges of motion, low back extensor muscles endurance and walking capacities preoperatively. Lessons learned from this pilot study will inform the design of a future phase III clinical trial. Further studies are necessary to assess the impact of physical exercise as a stand-alone intervention or as part of a multimodal approach to prehabilitation with patients awaiting elective spine surgery.

## Supplementary information

Trial protocol
